# Supplementary material for: Induction of Hepcidin Expression in the Renal Cortex of Sickle Cell Disease Mice
Source: Int J Mol Sci. 2023 Jun 28;24(13):10806. doi: 10.3390/ijms241310806 (PMC10341858; doi:10.3390/ijms241310806)
Supplement: Supplementary file 1 [file ijms-24-10806-s001.zip › ijms-2438249-supplementary.pdf]

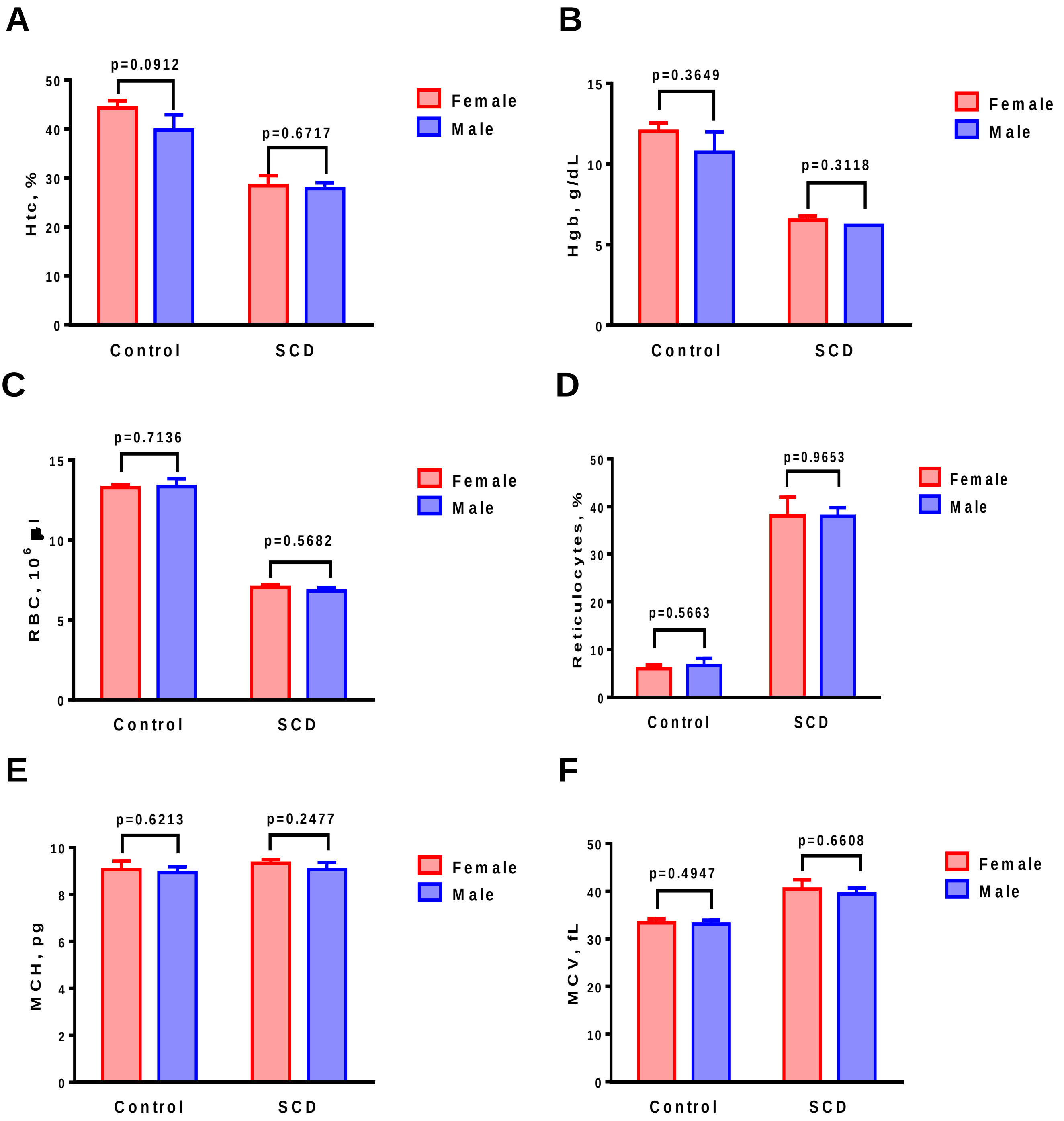

Supplemental Figure S1

Hematological paraments in male and female mice. No statistically significant differences are observed between male and female mice in the hematocrit (A), hemoglobin (B), red blood cells count (C), mean corpuscular volume (D), mean corpuscular hemoglobin (E) and reticulocytes (D) of the control and SCD mice. Blood and urine samples are collected from 3 males and 3 females in SCD and control groups. Hematological parameters are assessed using hematology counter Sysmex XN-1000 with C57BL/6 chip. Statistical analysis was performed using Prizm 6.0 software (GraphPad). Data was analyzed using 2-way ANOVA with the Turkey post hoc test. Means and STD are shown in the graphs. $p<0.05$ is considered significant.

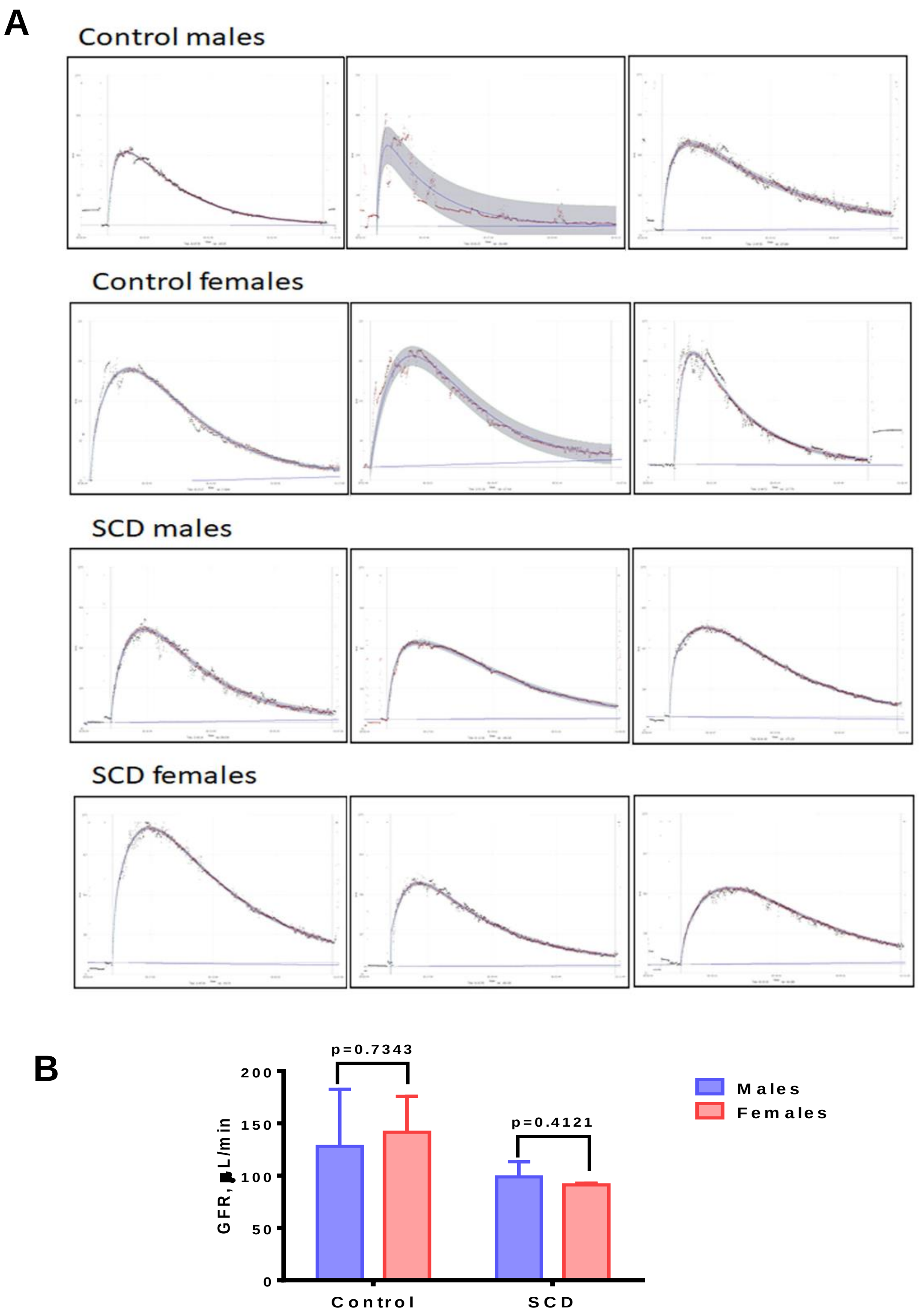

Supplemental Figure S2

GFR levels in male and female mice. No statistically significant differences are observed between male and female mice in the GFR values. (A) Raw data obtained from detection of sinistrin-FITC with fluorescent detector (MediBecon GmbH). (B) Quantification of GFR. 3 females and 3 males are used to in the control and SCD groups. Statistical analysis was performed using Prizm 6.0 software (GraphPad). Data was analyzed using 2-way ANOVA with the Turkey post hoc test. Means and STD are shown in the graphs. $p<0.05$ is considered significant.

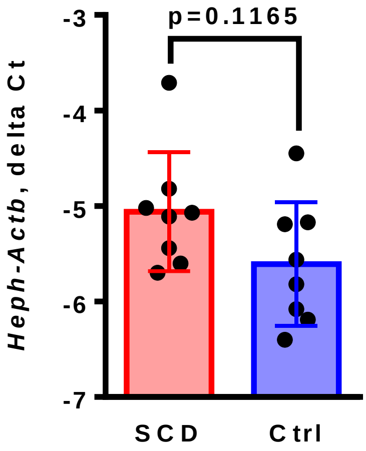

Supplemental Figure S3.
Expression of renal hephaestin (Heph) is similar in SCD and control mice.
Real time RT-PCR of mRNA isolated from renal cortex. Results are normalized to β-actin levels (*Actb*) and shown as delta Ct for mRNA. Means and STD are shown. Each dot represents a value obtained from an individual mouse. $p<0.05$ is considered significant.

Supplemental Figure S4. *Full size Western blot gels*
Loading order SCD1, SCD2, SCD3, SCD4, Ctrl1, Ctrl2, Ctrl3, Ctrl4

Fig. 2C TfR

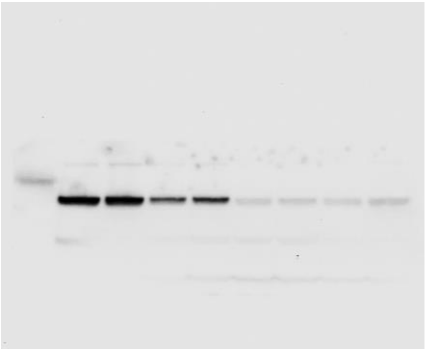

Fig.2C β-Actin

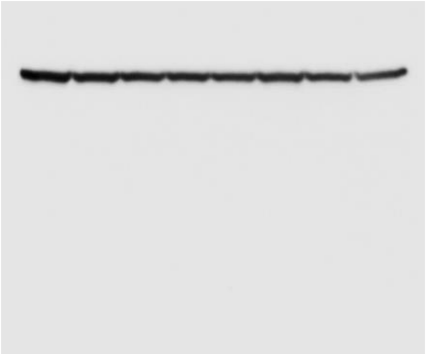

Fig. 2G FRTH

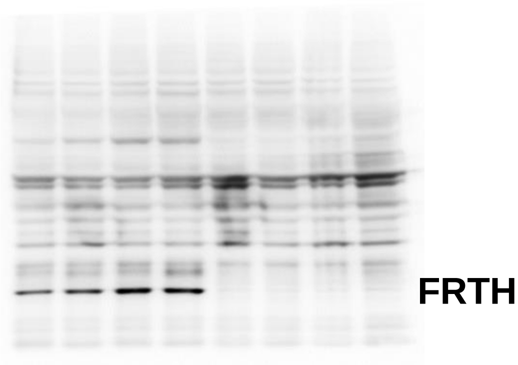

FRTH

Fig.2G β-Actin

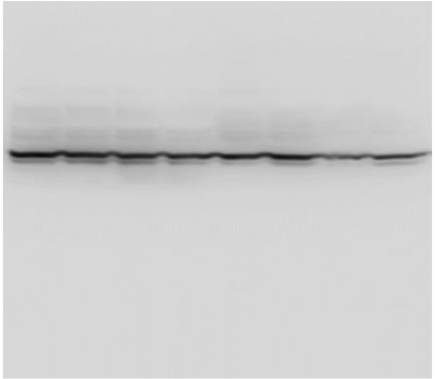

Fig.3B FPN

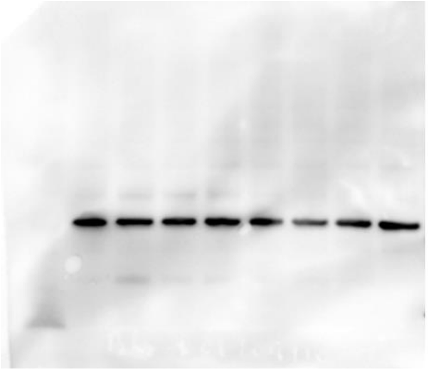

Fig. 3B β-Actin

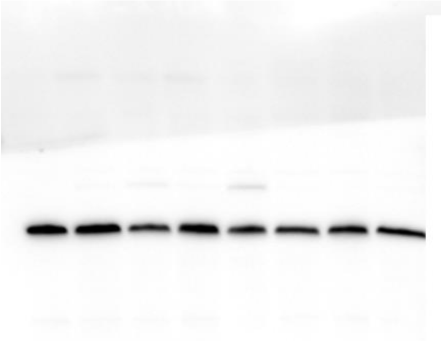

Fig.3E CP and β-Actin

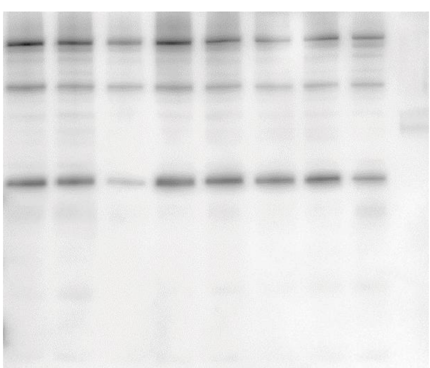

CP

Actin

Fig. 3H FPN and β-Actin

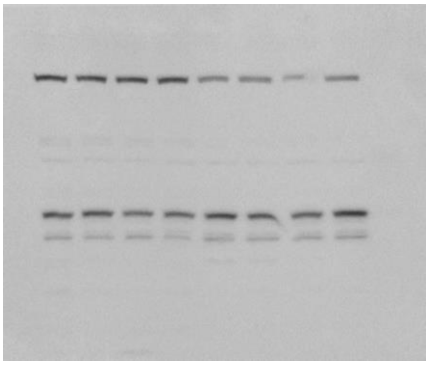

FPN

Actin
